# A quantitative literature-curated gold standard for kinase-substrate pairs

**DOI:** 10.1186/gb-2011-12-4-r39

**Published:** 2011-04-14

**Authors:** Sara Sharifpoor, Alex N Nguyen Ba, Ji-Young Young, Dewald van Dyk, Helena Friesen, Alison C Douglas, Christoph F Kurat, Yolanda T Chong, Karen Founk, Alan M Moses, Brenda J Andrews

**Affiliations:** 1 Department of Molecular Genetics, The Donnelly Centre for Cellular and Biomolecular Research, University of Toronto,160 College Street, Toronto, M3S 3E1, Canada; 2 Banting and Best Department of Medical Research, University of Toronto, 112 College Street, Toronto, M5G 1L6, Canada; 3 Department of Cell and Systems Biology, University of Toronto, 25 Harbord Street, Toronto, M5S 3G5, Canada; 4 Centre for the Analysis of Genome Evolution and Function, University of Toronto, 25 Willcocks Street, Toronto, M5S 3B2, Canada

## Abstract

We describe the Yeast Kinase Interaction Database (KID, http://www.moseslab.csb.utoronto.ca/KID/), which contains high- and low-throughput data relevant to phosphorylation events. KID includes 6,225 low-throughput and 21,990 high-throughput interactions, from greater than 35,000 experiments. By quantitatively integrating these data, we identified 517 high-confidence kinase-substrate pairs that we consider a gold standard. We show that this gold standard can be used to assess published high-throughput datasets, suggesting that it will enable similar rigorous assessments in the future.

## Background

Protein kinases constitute one of the largest protein families, accounting for approximately 2% of eukaryotic genomes. Kinases catalyze the transfer of phosphate groups to proteins, thereby influencing their activity, localization, stability, conformation and/or ability to interact with other proteins [1]. The yeast genome encodes 127 protein kinases, 20 of which are required for cellular viability [2,3]. At least 30% of the yeast proteome [4] is estimated to be phosphorylated, yet only a small portion of these phosphorylation events have been associated with their cognate kinase [5]. In fact, PhosphoGRID database (v.1.0) reported over 5,000 phosphorylation sites in 2010, amongst 1,500 proteins in both high-throughput (HTP) and low-throughput (LTP) datasets in yeast, 90% of which have not been associated with either a function or a regulatory kinase [6]. Since many phosphorylation events are highly transient or occur in the context of specific physiological conditions, it is difficult to capture kinase-substrate interactions. Furthermore, redundancy and promiscuity of protein kinases (particularly *in vitro*) can often complicate biochemical analysis.

Many targeted and HTP approaches have been used to link kinases and substrates in budding yeast, including: the use of analogue-sensitive kinase alleles for *in vitro *phosphorylation assays [7,8]; the interrogation of proteome chips with purified kinases to identify rosters of proteins phosphorylated *in vitro *[5,9]; affinity purification to discover kinase-associated proteins [10-13]; systematic genetic screens to identify genes that functionally interact with kinases [14-16]. Given the differences in the ability of large-scale datasets to capture kinase-substrate relationships and the number of different experimental approaches used to associate kinases with their targets, there is a requirement for both accurate quality assessment for HTP datasets through assembly of reliable gold standards and systematic data integration of information in the literature with HTP datasets.

Significant efforts have been made in this regard, including: PhosphoELM, a database of experimentally verified phosphorylation sites in all eukaryotic proteins [17,18]; PhosphoSite, a literature-curated database that compiles post-translational modifications with a focus on phosphorylation in all organisms [19]; NetworKIN [20], a database that integrates consensus substrate motifs of human kinases with *in vivo *phosphorylation sites, protein-protein interaction networks and kinase domain sequences in order to quantitatively predict cellular kinase-substrate relationships; and PhosphoGRID, which includes information from the literature on *in vivo *phosphorylation sites for all yeast proteins and assigns the appropriate kinase or phosphatase responsible for each phosphorylated residue [6]. All of these databases focus on consensus sites and phosphorylated residues. However, there is also considerable experimental information about kinase-substrate relationships at the protein level that is not easily represented in these databases. On the other hand, databases such as BioGRID [21,22], which stores all protein and genetic interactions, do not represent the additional specific biochemical experiments that are performed in order to determine kinase-substrate relationships.

We sought to systematically amalgamate interaction information from many experimental approaches - genetic, biochemical and physical - with the specific goal of defining a *bona fide *interaction between kinases and substrates. We reasoned that a database designed to compile a reliable gold standard for kinase-substrate interactions would require: 1) a means of distinguishing upstream and downstream interactors of kinases, kinase activators and regulatory subunits or co-activators and complex components; 2) a measure of the directionality of genetic interactions involving kinases (for example, suppression, dosage lethality and dosage suppression); 3) a means of including a quantitative measure of the significance of a biochemical interaction; 4) a method for producing a score that reflects the quality of the evidence in the literature supporting a kinase-substrate relationship.

To address these issues, we developed Yeast KID, the first literature-curated database for kinases that integrates a series of HTP and LTP, genetic, physical, and biochemical experimental evidence with the goal of establishing known kinase-substrate relationships. KID enables not only the assembly of tailored gold standards of kinase-target pairs, but also provides a ranked score for assessing the quantity and quality of evidence supporting each pair. KID features a user-friendly interface that amalgamates all genetic, physical, and biochemical HTP data involving yeast kinases, providing easy access for integrative analysis and more complex bioinformatic approaches to study kinase pathways.

## Results and discussion

### Database features

#### Content

**Figure 1:**
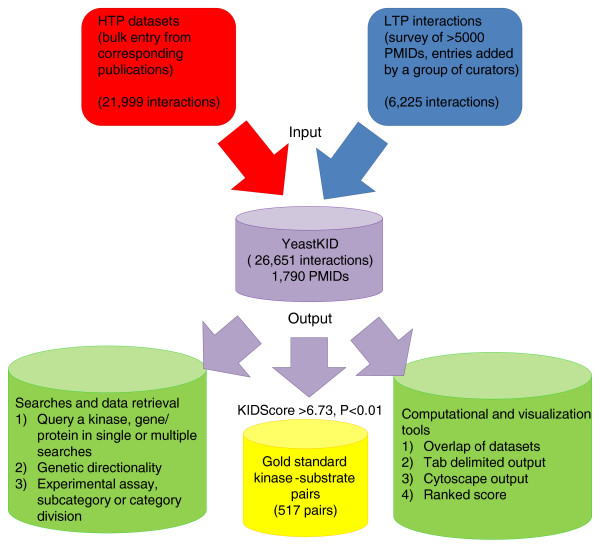
**Inputs and outputs of Yeast KID**. Organization of information in KID and key analytical tools are shown. The interface combines HTP and LTP information into a single database that can devise a score as an output for each interaction, in order to define the subset of gold-standard kinase-substrate pairs. Kinase interactions can be queried using KID by querying either genes or kinases as single or multiple searches. Excel and Cytoscape-compatible display and the ranked score simplify overlap analysis and data extraction.

Yeast KID reports interactions between 127 kinases (Table S1 in Additional file 1) and genes/proteins in a hierarchical manner (Figures S1 and S2 in Additional file 2). Entries are focused on experimental categories pertaining to substrate identification. LTP and HTP kinase interactions are combined in a single table format, based on 31 biochemical, physical, and genetic categories (Figures S1 and S2 in Additional file 2). For the purpose of Yeast KID, we define a kinase-gene interaction as any evidence that links a kinase to another gene or protein, which includes genetic, biochemical, physical or phenotypic experimental evidence. Table S2 in Additional file 1 shows the distribution of the number of kinase-gene interactions reported for each kinase in Yeast KID. The average number of unique interactors is 210, with a range from 883 for Slt2 and Bck1 to 16 for Rio1. The database includes 6,225 LTP and 21,990 HTP kinase-gene interactions, with 100% coverage of the kinome for HTP and approximately 85% coverage for LTP categories. With 108 LTP literature-curated kinases, Yeast KID reports high quality data compiled by our group after reviewing over 5,000 publications, with approximately 1,800 PubMed identifiers (PMIDs) entered into the database. Because multiple PMIDs may support a single kinase-gene interaction under the same category, KID contains over 35,000 entries in total. Curation guidelines were consistently followed to create a unified database (see Materials and methods; Figure 1). However, kinases of certain cellular processes are less represented in the LTP categories. For example, kinases of the mating pathway and DNA replication are highly under-represented, while most cell cycle regulatory kinases have been completely curated for LTP interactions in the latest version of KID.

#### Display

The KID database uses a web interface where kinases and their interacting genes/proteins are connected through a distinct PMID, displayed as a checkmark (Figure S1 in Additional file 2). Interactors and kinases are displayed in the first and second columns of the table, respectively, while the remaining columns represent experimental categories. The interface includes a color box (left side of display) that allows selection of interactions in one or more experimental categories (same color, OR) or overlapping interactions of two or more categories (different color, AND), with inclusion of additional categories (light green) or exclusion of specific categories (white). We incorporated AND logic for multiple color sets such that (blue OR blue) AND (green OR green) would select the overlap between all interactions selected in either blue category with any of the interactions colored in either green category (Figure S1 in Additional file 2). Each category can be singularly selected or removed, allowing for overlap analysis between datasets individually, or in combination. The complete dataset can be downloaded by clicking on the 'Search' button, without indicating any kinase, gene or KID score threshold in the score box. Definitions of all experimental categories and the functions of each button can be viewed directly on the site by clicking on a bubble icon close to each category or function.

#### Interface for queries and searches

We designed the KID interface to facilitate searches for a variety of interactions relevant to kinase biology. All searches can exploit the color box system to include multiple queries with specific experimental output displays, either individually or in combination (Figure S1 in Additional file 2). For example, all interactions pertaining to one or more kinases can be queried using the 'Search' button. Using this application, all interactions for all kinases in the query ID will be displayed in alphabetical order and with the relevant PMID. Overlapping interactions involving specified kinases or the kinases associated with a list of genes/proteins can be acquired using the 'Compute Kinase Overlap' or the 'Compute Gene Overlap' buttons, respectively.

The number entered in the 'Score' box in the KID interface determines the lower threshold of display. The score is a measure of the strength of evidence associating a kinase-substrate pair, and is arbitrarily set to -5 as the default (see below for more information about the KID score). We recommend using KID scores corresponding to a *P *< 0.01 (currently 6.73) for high quality kinase-substrate gold standards and *P *< 0.05 (currently 4.72) for less stringent lists of kinase-substrate pairs. KID automatically sorts the output interactions of a search from the highest to lowest scoring kinase-substrate pair, except for overlap searches involving multiple kinases or genes, as noted above. Each search creates tab-delimited (.txt) and Cytoscape-compatible [23] network files that can be downloaded for other forms of visualization. All evidence for each kinase-gene/protein pair is presented via a green checkmark that, when clicked, displays the PMID, first author information and more detailed curator notes (Figure S1 in Additional file 2). A unique feature of the KID interface is the capacity to perform detailed searches using specific experimental categories.

### The KID score

The variety of different experimental approaches used in defining a kinase-substrate pair creates a challenge in accurately associating a substrate with a particular kinase [24]. In fact, many kinase-substrate pairs are supported by a small number of experiments that are not usually consistent across kinases or substrates. For example, some proteins are difficult to purify for *in vitro *kinase assays, while other *bona fide *substrates fail to show a phosphorylation-dependent change in mobility following SDS-PAGE. Hence, there is a need for a quantitative approach that defines confidence in kinase-substrate pairs based on the quality and quantity of experimental evidence of different types.

One approach to combining experimental evidence is the sum of the total number of interactions, used in the unified database, BioGRID [21,22]. Also, BioGRID has recently reported a new scoring system, which assigns more value to physical rather than genetic interactions (1.5 points for physical and 1 point for genetic) [21,22]. Although generally useful, we reasoned that this approach may not be optimized for scoring kinase-substrate relationships for a number of reasons: 1) it is unclear whether the number of experiments supporting a particular kinase-gene/protein connection is a useful measure of whether a protein is an *in vivo *kinase substrate; 2) databases such as BioGRID include more general experimental categories in their curation method that apply to all genes, rather than specific phosphorylation assays, and may not be of sufficient specificity to accurately assess a kinase-substrate relationship; 3) a larger weight for physical rather than genetic interactions may not be appropriate for the typically transient physical interactions associated with kinases and their substrates [25].

We addressed these issues in Yeast KID by including a hierarchical classification of experimental categories, specifically designed to be relevant for kinase-substrate interactions (Figure S2 in Additional file 2). Using a positive training set of well-defined kinase-substrate pairs, we computed log-likelihood ratios that summarize the weight for each experimental category (Table S3 in Additional file 1, Figure S3 in Additional file 2; see Materials and methods). These weights are then summed to give a KID score that represents a measure of the strength of existing evidence in the literature supporting a kinase-substrate relationship. The weight of each experimental category will change as more interactions are entered (Figure S3 in Additional file 2).

Based on the data currently in KID, most HTP categories had a small but significant contribution to the KID score, except for the *in vitro *phosphorylation category, which made a large contribution. This bias likely reflects large datasets describing *in vitro *targets for the well studied Pho85 and Cdc28 cyclin-dependent protein kinases (Cdks), which have been surveyed for *in vitro *substrates using analogue-sensitive alleles [7,8]. By contrast, many LTP categories performed well in identifying kinase-substrate pairs from our training set, with the highest scoring categories being *in vitro *kinase assays, site-directed mutagenesis, *in vitro *phosphorylation site mapping and phospho-shifts, all biochemical assays of the enzymatic activity of a kinase. However, no single category contributes sufficiently to the score to call a kinase-target pair at the stringent cutoff, which reflects the intuition of experts that no single currently available experimental method is sufficient to conclusively define kinase-substrate relationships.

**Figure 2:**
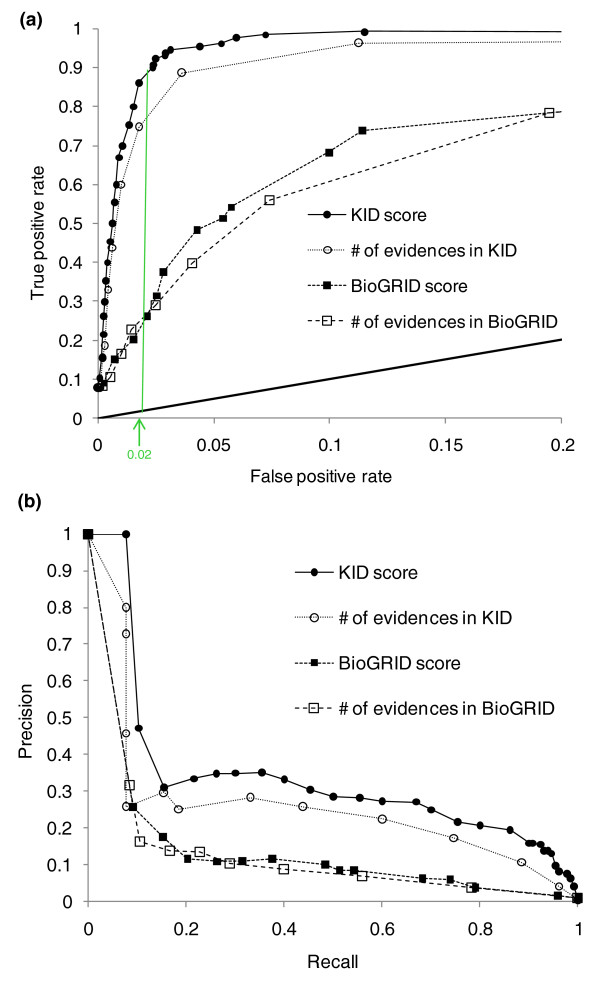
**Yeast KID performance in identifying kinase-substrate pairs**. **(a) **The graph indicates the true positive rate detected by the number of experimental evidence supporting a kinase-substrate interaction reported in KID and BioGRID [21,22] and the top scores reported in both databases for kinases, as a function of their false positive rates (ROC curve). The diagonal line represents the random assignment of positive classes. The green line shows the cutoff score used for the stringent gold standard of kinase-substrate pairs. **(b) **The precision of the number of experimental evidence supporting kinase-substrate interactions and the respective top scores reported in KID and BioGRID [21,22] for yeast kinases, as a function of their recall (equivalent to the true positive rate). The performance of a random assignment of positive classes is not shown as it is too low for representation.

To test the capacity of the newly defined KID score to identify known kinase-substrate pairs, we performed a ten-fold cross-validation (Table S3 in Additional file 1). For this cross-validation, we separated the data into ten bins. For each cross-validation step, a single bin was used as the test set while the other nine were used to estimate the weights for each category. To understand the trade-off between sensitivity and specificity, we computed a receiver operating characteristic (ROC) curve (true positive and false positive rates of each method at different thresholds; Figure 2a). The predictions for every test set in each of the cross-validations were summed to produce the final curve. At a set false positive rate, multiple true positive rates can be obtained and we display the worst true positive rate (Figure 2). For additional clarity, we have removed additional points by only displaying the maximal false positive rate at intervals of true positive rates.

To compare the KID score to other possible scoring schemes, we compared the performance of the following scoring methods in predicting kinase-substrate pairs using the positive training set: 1) the number of interactions reported in BioGRID [21,22]; 2) the BioGRID general scoring scheme [21,22]; 3) the number of interactions reported in Yeast KID; and 4) the KID score. Performance was tested by calculating ROC and precision-recall curves (Figure 2a, b). We note that the precision appears low, but the expected precision of a random classifier in these data is <1 × 10^-4^. This analysis shows that, by using a score based on the phosphorylation-specific and more detailed curation, Yeast KID performs better at identifying the positive training set (Figure 2). Specifically, BioGRID identified 25% of kinase-substrate pairs in our positive training set, while KID identified 90% of known targets (< 2% false positive rate). Furthermore, while counting the number of interactions in KID performs moderately well, the KID score is still more sensitive in identifying a kinase-substrate pair against a set of random pairs.

We next plotted the top kinase-interacting pairs reported in BioGRID against the top pairs reported in Yeast KID, to ask whether the same pairs were identified. While there was overlap amongst the top interactions in both databases, the two scores identified distinct kinase-interacting pairs; 248 interactions were shared amongst the top 517 pairs in both databases. Together with the higher predictive performance of the KID score (Figure 2), we conclude that the top scoring interactions in Yeast KID most likely identify a more confident gold-standard set of protein kinase-substrate interactions than could be identified from more general interaction databases.

We note that the KID score is highly dependent on the initial positive training set and only calculates the likelihood that an interaction belongs to the initial positive training set as opposed to a random pair from our database. In theory, the larger and the more accurate the initial positive training set, the more confidence we have that the score accurately reflects the strength of evidence supporting a kinase-target pair. We carefully chose the positive training set by including over 120 kinase-target pairs representing 20% of the stringent gold-standard pairs reported in this study, with coverage of 40 kinases in order to minimize the scoring bias. However, the score cannot account for internal biases in published experiments. For example, more labor-intensive assays are obviously less represented in publications. Also, there may be a bias for well-studied kinase-target pairs. In a similar vein, interactions that are tested but result in negative outcomes are often not represented in publications and are not curated. Also, inevitable inconsistency in coverage of data from each publication during the curation process may contribute to variability in the KID ranking. Thus, the KID score displays a relative rather than absolute ranking, which is dependent on the initial positive training set (Table S3 in Additional file 1). Finally, the KID score is most likely a conservative measure for evidence supporting a kinase-substrate pair because uncharacterized kinase-target pairs may exist among the kinase-substrate interactions that we assume to be negative. This means that the KID score is most likely an underestimate of the strength of evidence supporting a kinase-substrate pair in comparison to random pairs in the space of all possible interactions.

### Applications

#### Defining a gold-standard kinase-substrate set using KID scores

**Figure 3:**
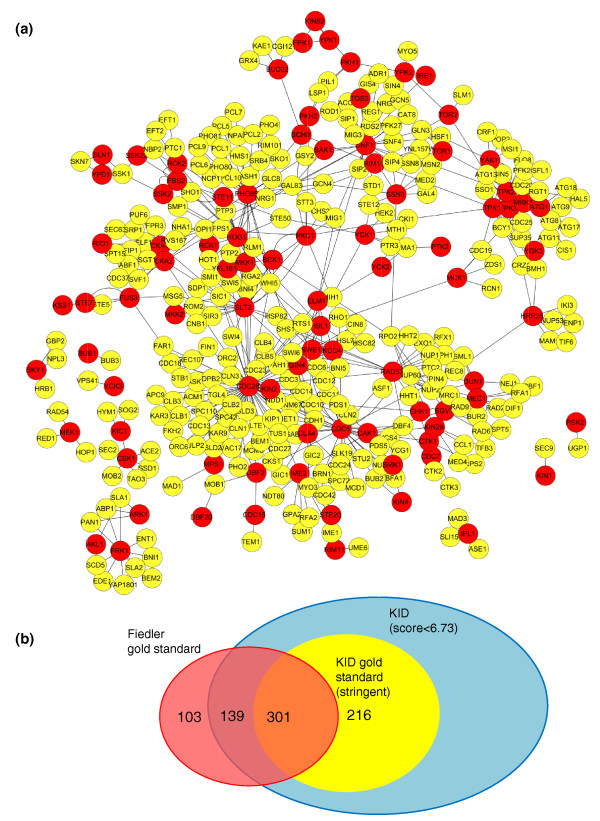
**Literature-curated gold-standard kinase-target pairs predicted by KID**. **(a) **Spring-embedded edge-weighted Cytoscape network [23] showing the gold standard for kinase-substrate pairs. Kinases (red nodes) are connected to their targets (yellow nodes) using the KID score as the strength of the interaction (edges). The network includes 517 pairs at the stringent KID score cutoff of 6.73 (*P *< 0.01). **(b) **Comparison of KID gold standard with published gold standard for kinase-substrate interactions [15]. The stringent KID gold standard is depicted in yellow while the gold standard published in Fiedler *et al*. [15] is shown in pink. The number of interactions that overlap are indicated on the diagram. The large blue circle includes all the more than 26,000 entries in KID, but only 517 represent the gold standard.

We used the calculated KID score to compile a ranked list of 517 kinase-substrate pairs (stringent KID score cutoff of 6.73; false positive rate < 2%), which we define as the 'gold-standard' pairs of kinase-substrate interactions. At this cutoff, the KID score performs significantly better than the binary BioGRID score in identifying known positive training set kinase-substrate interactions (90% versus 25% of true positives at the same false positive rate; Figure 2). The gold standard defines a highly connected network of kinase-substrate interactions with a bias towards well-studied cell cycle regulatory kinases, Cdc28 and Pho85, and the polo-like kinase Cdc5 (Figure 3a; Figure S4 in Additional file 2). This bias likely reflects several factors: the availability of large scale datasets for *in vitro *substrates of Pho85 and Cdc28 [7,8]; the importance of phosphorylation as a mechanism of cell cycle regulation (90 targets with 222 phosphorylated residues reported in PhosphoGRID [6]); and over-representation of experiments on biologically predominant kinases, such as cyclin-dependent kinases in the yeast literature. There were six substrates on average for each kinase in our gold standard, ranging from 70 reported targets of Cdc28 [26] to no clear substrates for 37 kinases.

We next compared the quality of our gold standard to a recently compiled list of yeast kinase-substrate pairs used to analyze genetic interaction data, obtained from assessing genetic interactions between kinases, phosphatases and selected regulators [15]. Results of this analysis are depicted as a Venn diagram in Figure 3b. The two lists overlapped by 58% (301 kinase-substrate pairs) while 139 pairs scored too low in KID to be considered a gold-standard kinase-substrate pair. We failed to identify 103 interactions in the Fiedler *et al*. [15] standard during our curation process. Since PMIDs were not reported for this dataset, it was difficult for us to reconcile these results. There were 123 interactions in Fiedler *et al*. [15] that belong to the 19 kinases that were not curated for LTP interactions in KID. The low overlap between the two gold standards highlights the importance of systematic curations in conjunction with appropriate scoring schemes in defining a useful benchmark for quality assessment of HTP datasets.

The KID score quantification can be used to rank targets of kinases that fall below the stringent cutoff. For example, many kinase-gene pairs that we curated fall below our stringent cutoff, but the relationship is supported by many lines of evidence. Further characterization of the candidate genes with high KID scores (through complementary experimentation according to pre-existing data in KID) may confirm novel targets of kinases. Since the KID score is a relative ratio for kinase-gene/protein pairs and provides a ranking scheme, it can predict the likelihood that one gene is regulated by one kinase versus all other kinases. The KID provides a means to quantify literature-curated evidence connecting kinases and other proteins for target prediction.

#### Comparison of HTP assays in the coverage of interaction space and in identifying gold standard kinase-substrate pairs

One important application of a kinase-gold standard is assessment of the quality of HTP datasets. Recently, a systematic comparison of HTP and LTP experiments using physical interaction data as a test case [27] revealed that HTP physical interaction datasets are comparable in quality to their LTP counterparts. We performed a similar analysis comparing HTP and LTP kinase interaction data from each of physical, genetic, biochemical and localization experiments curated in Yeast KID, both individually and as a whole.

##### Overlap between genetic, biochemical and physical interaction datasets

**Figure 4:**
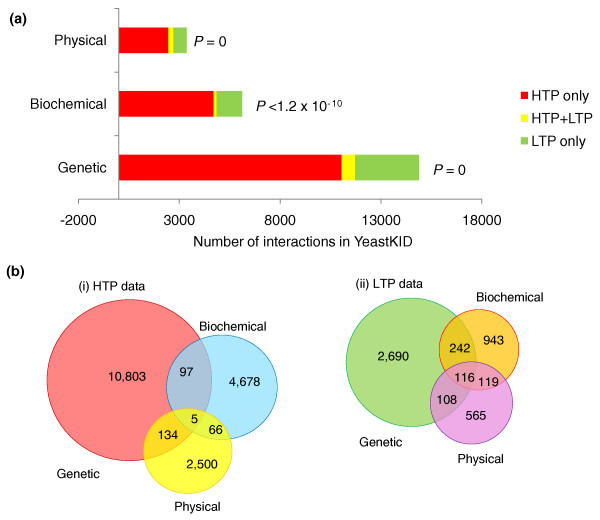
**Comparison of the relative amounts of genetic, physical and biochemical HTP and LTP data for kinases**. **(a) **Bar graph illustrating the relative amounts of genetic, physical and biochemical interactions in HTP, LTP and both data forms. *P*-values indicate significance of the overlap in a given interaction space. **(b) **Overlap of all three assays in HTP and LTP methods. LTP data show the largest overlap between genetic, physical and biochemical approaches, while HTP data show little overlap.

We first assessed the quality of existing HTP data in identifying their relevant LTP interactions curated in KID. In general, HTP phosphorylation datasets were enriched for phosphorylation targets detected by LTP assays. Particularly, *in vitro *phosphorylation assays using analogue-sensitivity alleles [7,8,28] and HTP assays indicating general *in vivo *dependency on a kinase were highly enriched for proteins identified by an equivalent LTP assay (Figure S5 in Additional file 2). Both HTP physical interaction and genetic interaction datasets were also enriched for interactions found by a LTP assay of the same type, although the HTP physical interaction data performed slightly better in this test. (Figure S5 in Additional file 2). We reason that this difference may largely reflect the relative size of each dataset. Genetic interaction datasets are approximately ten-fold larger than HTP protein interaction datasets (with over 11,000 interactions), while the amounts of data for genetic, biochemical and physical assays in the LTP literature are comparable (Figure 4a). By contrast, HTP co-localization studies showed no overlap with LTP co-localization (Figure S5 in Additional file 2). While LTP co-localization studies define the localization of two differentially marked proteins simultaneously, we defined HTP co-localization if two proteins were localized to the same subcellular compartment, excluding all cytoplasmic and nuclear data [29].

Despite the high enrichment of HTP genetic, physical and biochemical assays for LTP data of the same type, many LTP interactions were not captured by the HTP methods, suggesting that HTP and LTP datasets generally have different coverage of the interaction space. The lack of overlap may also reflect the technical nature of HTP assays, which typically survey all kinases under the same conditions, rather than directed approaches, which involve experiments functionally tailored to the kinase of interest. Only a handful of genes were present in all three sets of HTP data, suggesting differential coverage by the three types of HTP data as well (Figure 4b). While LTP data had more overlapping pairs between genetic, physical and biochemical assays, the reported data comprise only a fraction of the total data present in the literature.

##### Assessment of all HTP datasets in identifying the KID gold-standard set

**Figure 5:**
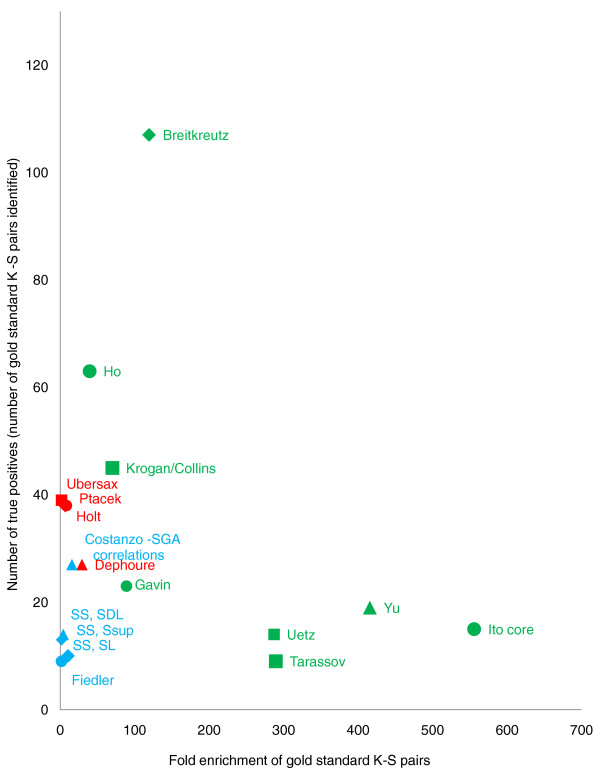
**Relative performance of HTP datasets in identifying known kinase-target pairs**. Number of gold-standard kinase-substrate pairs identified in each dataset is plotted against the fold-enrichment of kinase-substrate (K-S) pairs found in each dataset. Red, biochemical; green, physical; blue, genetic. See text for details. SDL, synthetic dosage lethality; SGA, synthetic genetic array; SL, synthetic lethality; SSup, synthetic suppression. SS refers to the source for the SDL, SL, SSup data reported in Sharifpoor *et al *(Functional wiring of the yeast kinome revealed by global genetic network motif analysis, submitted).

We used the KID gold-standard set to test the relative ability of each individual HTP dataset to identify kinase targets. We computed the enrichment of gold-standard kinase-substrate pairs identified by each dataset (which we defined as true positives for this analysis), considering the number of interactions tested for each dataset (Figure 5; see Materials and methods). The most informative dataset in terms of both the number of kinase-substrate pairs identified and the fold-enrichment in the gold standard was a recent survey of protein-protein interactions involving kinases identified by a modified protein pull-down approach in combination with mass spectrometry [13]. Yeast two-hybrid datasets were also highly enriched for kinase-substrate pairs [30,31], but identified far fewer targets (true positives) than the protein-protein interaction datasets [10,11,13,32].

Overall, phosphorylation and physical interaction datasets performed better than genetic interaction datasets in identifying the KID gold-standard kinase-substrate pairs. Although correlations of genome-wide genetic interaction profiles (synthetic genetic array (SGA) correlations) [16] and HTP synthetic dosage lethal (SDL) screens (Sharifpoor *et al*: Functional wiring of the yeast kinome revealed by global genetic network motif analysis, submitted) are enriched for gold-standard kinase-substrate pairs, other genetic datasets alone are not informative in defining these relationships [15]. Since kinase-substrate relationships involve a direct physical interaction, it stands to reason that biochemical and physical interaction assays are more likely to directly identify links between kinases and their targets. Also, genetic interaction datasets are currently largely populated with synthetic lethal interactions, which often identify genes that function in parallel pathways, and not substrates in the same pathway [16].

### Clustering kinases based on their functional targets

While yeast kinases have been previously classified based on their sequence similarity [2,33], there has been no systematic attempt to quantitatively classify kinases based on their targets. Since KID scores are relative across all kinase-gene/protein pairs, we reasoned that by calculating the correlations of all kinase pairs, we could functionally classify groups of kinases involved in similar processes based on their targets. We used only binary values to calculate correlations between the kinases in the gold standard in our analysis; two kinases were correlated if they shared the same target(s). Therefore, the correlation analysis considers only the most confident targets of a kinase, rather than all possible targets. The magnitude of the KID score was not used for correlation assessment.

**Figure 6:**
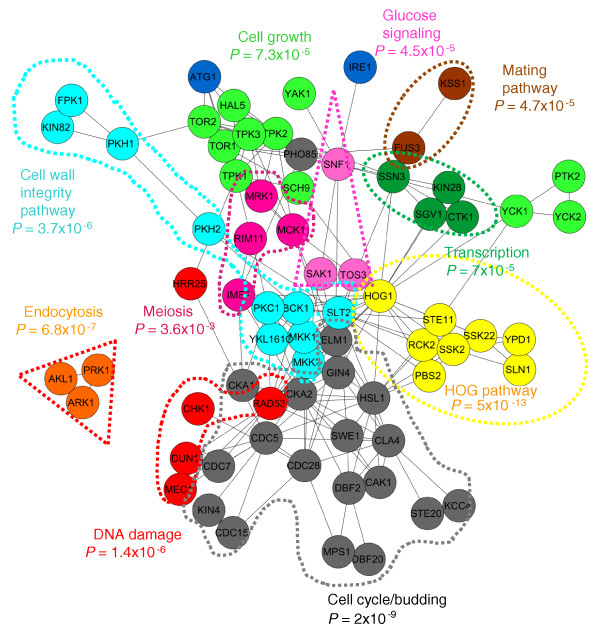
**Functional classification of kinases in the gold standard based on target overlap**. Cytoscape edge-weighted force-directed diagram plots the correlation of kinases curated in the KID gold standard (nodes), based on similarity of their targets (depicted as edges corresponding to correlation scores). Kinases that have multiple overlapping targets are more correlated and cluster together in the network. Spatial organization of the nodes in the network classifies kinases based on their shared interaction profile. *P*-values indicate enrichment of Gene Ontology function using FunSpec [41]. Nodes in the same functional group are depicted as similar colors. Blue nodes represent correlated kinases that do not fall into any functional class. Of the 87 kinases present in the gold-standard kinase-substrate pairs, 71 share at least one target with one or more kinases. HOG, high osmolarity glycerol.

We display the results of our analysis as a network diagram that describes the subcategories of kinases in the gold standard based on their targets (Figure 6). The edges (weighted by binary correlations) estimate the relative overlap of two kinases (represented as nodes) in regulating the same cellular substrates. The highly connected network shows that most kinases in the gold standard share at least one target with another kinase. Furthermore, the diagram illustrates the complex buffering of kinase pathways, particularly in the cell cycle group, since most kinases are highly correlated with several overlapping targets. Spatial organization of the groups of kinases suggests a cellular model whereby the cross-talk between different cellular processes is mediated through specific kinases (Figure 6). Results from the clustering analysis suggest a complex model that agrees with recent findings in a large-scale kinase proteomic study highlighting the complex interplay between kinase pathways [13].

Our correlation analysis discovers known functional relationships involving kinases. For example, the organization of the network suggests that the Snf1 kinase links transcription to glucose signaling, consistent with the well-established role of Snf1 in regulating transcriptional repression at promoters of genes required for growth on non-fermentable carbon sources [34-36]. Also, the network revealed multiple links between the high osmolarity glycerol pathway (required for growth on osmotic stress) and the cell wall integrity pathway, consistent with the high level of cross-talk known to occur between the two regulatory pathways *in vivo *[37]. For example, multiple genes show dependency on both the Slt2 and Hog1 kinases that regulate cell wall integrity- and high osmolarity glycerol-responsive genes, respectively. Our functional analysis shows that they also share multiple targets and corroborates previous reports that suggest a model whereby Slt2 phosphorylation is dependent on the Hog1-activating kinase Pbs2 [38-40].

We next tested whether highly correlated kinases are more likely to be functionally involved in the same biological processes [41]. We plotted an edge-weighted network diagram of all correlated kinases and searched for functional similarity of nodes within a proximal cluster using Gene Ontology terms (Figure 6). To define subclusters for functional analysis, we reorganized the network, placing each node in the closest subcluster based on correlation values. We saw that the calculated correlations are an excellent measure of functional similarity for kinase pairs (Figure 6), defining specific functional categories, further confirming that our ranking system is a valid relative score for functional kinase-substrate pairs.

## Conclusions

KID can be used to assess and compare the quality of new HTP approaches in identifying kinase-substrate pairs, LTP interactions of the same type, and the overlap with other HTP and LTP approaches. In addition, using the sophisticated search functions, filtering methods and user-friendly outputs, KID will provide a universal search system and repository for all datasets in HTP and LTP literature pertaining to yeast kinases.

## Materials and methods

### Database content

We defined 31 different types of experimental evidence relevant to defining kinase targets in a hierarchically classified format, first by high-throughput or low-throughput categories, then subclassified by physical, biochemical, genetic and phenotypic evidence (Figure S2 in Additional file 2). Data inputted for all HTP categories were extracted in bulk from the corresponding publications, while the LTP evidence, pertaining to specific phosphorylation assays, was inputted directly by a group of expert curators (Figure 1).

We extracted relevant articles for each individual kinase from PubMed by historically searching through every article published pertaining to the query kinase. We then compared our information with data from BioGRID, to extract additional publications that may have been missed during our curation process. Over 5,000 publications were surveyed up to August 2010 for LTP kinase interactions and all entries were inputted with the corresponding PMIDs. Curations were also performed based on definitions for the experimental evidence described on the website under each specific category (Figure S1 in Additional file 2).

### Curation process

Bidirectional interactions (for example, physical interactions, synthetic lethal interactions) were entered in both directions, while unidirectional interactions (for example, biochemical interactions, synthetic suppression) were only entered where a phenotype was clearly linked to a specific kinase. Evidence for interactions between kinases and other genes or proteins was entered with associated PMIDs ('kinase-gene interaction'), including the first author and year of publication. Directionality was added as notes where required (for example, dosage lethality) and specific allelic interactions and experimental design were also described in more detail in the notes section by the curator. Biochemical data regarding upstream regulators of kinases was not curated. If data pertaining to a conclusion were not shown in the publication or supplementary material, the evidence was not considered valid for entry into the database. Where there was more than one publication supporting the same interaction, each PMID was entered separately. For cyclin-dependent kinases with multiple regulatory subunits, the associated cyclin was also curated if specified in the literature. Each curator was supplied with detailed guidelines to maintain consistency and was assigned a set of kinases for literature curations. However, in the event that a publication included information for more than one kinase or between a kinase-pair, data were entered in KID for all kinases from a single paper to minimize curation errors through internal cross-checks.

### Quality assessment for each experimental category and definition of KID scores

To assess the quality of each individual experimental category in identifying kinase-substrate pairs, we used a simple scoring method that evaluates the likelihood that a category of interest identifies a true kinase-substrate pair as opposed to a false positive. We assembled a positive training set of kinase-substrate interactions, chosen by the curators based on the following criteria from low-throughput literature: 1) a defined physical interaction between the kinase-substrate pair; 2) the ability of the kinase to phosphorylate the substrate *in vitro*; 3) the ability of the kinase to phosphorylate the substrate *in vivo*; and 4) whether the site or effect of the phosphorylation event was known (Table S3 in Additional file 1). The positive training set includes 121 interactions for 40 kinases and is not biased for any particular experimental category. We compared the frequency of interactions in each experimental category to the frequency expected in a negative training set, which we defined to be kinase-protein interactions that are unlikely to represent *bona fide *kinase-substrate interactions. To do so, we had to compute the frequency of an experimental data point in each category in a set of proteins that are not substrates. Because we rarely know the proteins that are not substrates of a particular kinase, defining a negative set is a challenge. To obtain the number of experimental data points, we conservatively used all the experimental data found in KID that were not part of the positive training set. To compute the relative frequency in the negative training set, we needed to divide this value by the size of the negative training set. In principle, this would be the total interaction space (all kinases multiplied by all genes) minus the set of all *bona fide *protein-kinase substrate interactions. In practice, however, most datasets do not sample the entire interaction space (for example, HTP *in vitro *kinase assays) and the negative set must be normalized to reflect this. Therefore, we considered the HTP negative training set size to be a fifth of the total interaction space (one-fifth times the number of kinases multiplied by the number of genes). The negative training set size for the LTP categories must also be adjusted using the same rationale since LTP experiments have sampled even less of the entire interaction space. To determine the size of the LTP negative training set, we calculated the ratio of the number of interactions shown by LTP experiments to the number of interactions shown by HTP experiments, and reduced the negative training set size for the LTP categories by this ratio (HTP negative training set multiplied by the ratio). Therefore, we are assuming that HTP and LTP experiments have equivalent power to detect kinase-substrate interactions, but that HTP experiments explore a much larger space. By performing these adjustments on the negative training sets, we believe that the score represents a relatively unbiased measure of enrichment of the success of each category in identifying our positive training set.

The weight for each category is defined as the log ratio of the frequency of a particular category of experiment supporting a kinase-substrate pair from the positive training set compared to the negative set. For example, if a particular experimental category identified 50% of the positive training set but 10% of the negative training set, then the score for this category would be approximately the log of (50/10). We represent the positive training set as the matrix G, where G_j, i _= 1, if the i^th ^experimental category reported an interaction between the j^th ^kinase-substrate pair. The negative training set is similarly defined as R_j, I_, where R is either the HTP or LTP negative training set. The score is therefore:

where N_R _and N_G _are the sizes of the positive and negative training sets discussed above. One (1) count was added to each category as a pseudo-count in the positive set. In the negative set, N_R_/N_G _was added as a pseudo-count, to ensure that the ratio of experimental observations to a set size was the same in the positives and negatives, S_i _= 0 for that category. A similar likelihood ratio was recently used by Yu *et al*. [27], without the pseudo-count or a normalized negative training set. For the j^th ^putative kinase-substrate pair, the KID score is defined as:

where x_i, j _= 1 when the i^th ^experiment was reported for the j^th ^putative kinase-substrate pair.

In order to calculate *P*-values for the scored interactions, we randomized the evidence in each experimental category in the database and scored the randomized database. The resulting score distribution was used to obtain *P*-values.

In Figure 2, for the ROC curve with BioGRID, we have only considered positives that were present in either dataset when calculating the true positive rate.

In Figure 5, although the fold enrichment for each dataset is similar to our scoring scheme, no estimate of the sampled interaction space is required because most datasets indicate the number of tested interactions, except for physical interaction data collected using mass spectrometry techniques, for which we assumed full coverage. The negative training set size has been adjusted to match their reported interaction space coverage (number of tested kinases multiplied by number of tested genes). We note that the scoring for each experimental category is an estimate while the enrichment for each dataset is exact.

### KID schema

Figure S6 in Additional file 2 summarizes the overall schema for KID, which has a back-end and front-end composition. The back-end is managed through an in-house user control panel administrated by multiple curators. Curators use a relational database schema that enforces consistent entries, such that each individual can automatically observe previous entries by other curators for any kinase-gene/protein pair. The system allows for direct modification, removal or addition of more experimental evidence and internal cross-validation by curators. Curated interactions are then compiled in a single interaction table that, upon data entry or modification, is used to automatically calibrate the score function for each category and to generate whole database backups. Also, each curator modification is automatically logged for administrative purposes. The front-end of the database queries the relational database schema via Ajax to allow rapid feedback of requested information. The query system allows the whole database to be filtered based on multiple entries in various combinations (Figure S1 in Additional file 2). The query is then parsed by the server to identify the requested set of interactions, which are in turn directly displayed by the KID interface. This generated output can be downloaded as a tab-delimited copy or Cytoscape-compatible network file, or directly displayed as an interaction network using Cytoscapeweb [42]. A list of all database interactions can be viewed in Additional file 3.

### Correlations of kinases based on their targets

We compiled the targets of all the kinases within our gold standard (stringent cutoff) and performed an all-by-all comparison using Pearson's correlation coefficient. The correlation cutoff represents a *P*-value of 0.05 in the *t*-test statistics. Results from the correlation comparisons were then subjected to a graphical analysis using an edge-weighted scheme in Cytoscape [23]. Functional enrichment analysis was performed using FunSpec, a web-based cluster interpreter for yeast [41].

## Abbreviations

HTTP: high-throughput; KID: Kinase Interaction Database; LTP: low-throughput; PMID: PubMed identifier; ROC: receiver operating characteristic.

## Competing interests

The authors declare that they have no competing interests.

## Authors' contributions

SS, ANNB, BJA, and AMM designed the experiments and prepared the manuscript. SS, ANNB, JY, DVD, HF, ACD, CFK, YTC, and KF contributed to the literature curation process. SS and ANNB designed the interface and display. ANNB and AMM implemented the scoring scheme. All authors have read and approved the final version of this manuscript.

## Supplementary Material

Additional file 1 **Supplementary tables**. Table S1: kinases in Yeast KID. The list of kinases was compiled from the review by Rubenstein and Schmidt [2]. Kinases highlighted in blue were not curated in full. Table S2: distribution of kinase interactions in Yeast KID. Of the 127 kinases in budding yeast, all have been curated for HTP and 108 have been curated in LTP categories in Yeast KID, with the remaining 19 in progress (highlighted in blue). The mitogen activated protein kinases (MAPKs) have the highest number of interactions, whereas less characterized kinases (Rio1) have only a few interactions inputted. Table S3: positive training set of curated kinase-substrate pairs. List of *bona fide *kinase-substrate pairs defined based on curator's consensus. PMIDs for all pairs and the type of interactions used for selection are shown. Click here for file

Additional file 2 **Supplementary figures**. Figure S1: Yeast KID user interface. A screen-shot of the Yeast KID homepage is shown. Experimental categories are hierarchically displayed and queried individually or in combination using the color box (left). Kinases, genes/proteins or PMIDs can be queried either individually or in combination, as single or multiple genes/proteins separated by commas or spaces. For multiple queries, overlapping interactions can be searched using the 'compute gene overlap' and 'compute kinase overlap' functions. Definition of each category and function is displayed by clicking on the small bubble icon for each category. See text for details. Figure S2: hierarchical division of Yeast KID categories. Chart showing 31 experimental categories hierarchically organized in three levels: 1) HTP and LTP categories (green); 2) overall subdivision of genetic, phenotypic, chemical, physical, cell biological or biochemical approaches (blue); 3) specific experimental assays (purple). Figure S3: KID weights of different LTP and HTP experimental categories. Relative contribution of different experimental categories in identifying the positive training kinase-substrate set. The bar graph indicates the contribution of each category to the KID score. Bars highlighted with a red star show significance when comparing categories relative to a random assignment of positive classes. The total number of interactions entered in each KID category is also presented. Red, genetic; pink, physical; blue, biochemical; yellow, phenotypic; purple, cell biological; orange, chemical. Figure S4: distribution of kinase substrates in Yeast KID. The graph shows the distribution of kinase targets reported in Yeast KID at the stringent cutoff (*P *< 0.01). Cdc28, Cdc5, Snf1 and Pho85 kinases have the largest number of targets in the literature. Thirty-seven curated kinases have no targets in Yeast KID at the stringent cutoff and are not represented on the graph. Figure S5: assessing the quality of HTP datasets in identifying LTP interactions of the same type. Overlap of reported HTP interactions with the respective LTP interactions of equivalent assays. HTP assays enriched for their LTP counterparts are shown in bold. *P*-values indicate significance. Figure S6: KID schema. The back-end is managed through a customized user control panel that uses a relational database schema to enforce consistent entries. Curated interactions are compiled in a single interaction table that is used to calibrate the contribution score for each category and the overall KID score. Whole database backups are also generated, including logged tracking of curator modifications. The front-end of the database queries the relational back-end schema via Ajax, allowing rapid feedback of requested information. The customized query system (which allows for multiple inputs) is then parsed by the server to find the appropriate interactions to display on the KID interface. KID output can be downloaded in three different formats for further data manipulation. Click here for file

Additional file 3 **List of all database interactions (August 2010 update)**. Click here for file
