# Supplementary material for: A quantitative literature-curated gold standard for kinase-substrate pairs
Source: Genome Biol. 2011 Apr 14;12(4):R39. doi: 10.1186/gb-2011-12-4-r39 (PMC3218865; doi:10.1186/gb-2011-12-4-r39)

Supplementary Figure 1:

Colour box used to select specifc experimental categories of interest to be displayed using AND/OR functions

Searches interactions of a gene/ protein or multiple query genes /proteins with kinases, separated by space or comma

Searches for a common kinase or kinases interacting with two or more genes

Searches interactions of a kinase or multiple query kinases separated by space or comma or the complete database when no entires exist

Searches for a common gene /protein interacting with two or more kinases

Searches for all interactions curated from a single or multiple PMIDs separated by a space or comma

Describe the definition of each experimental category and search function when clicked on

Determines a threshold for KIDscores displayed

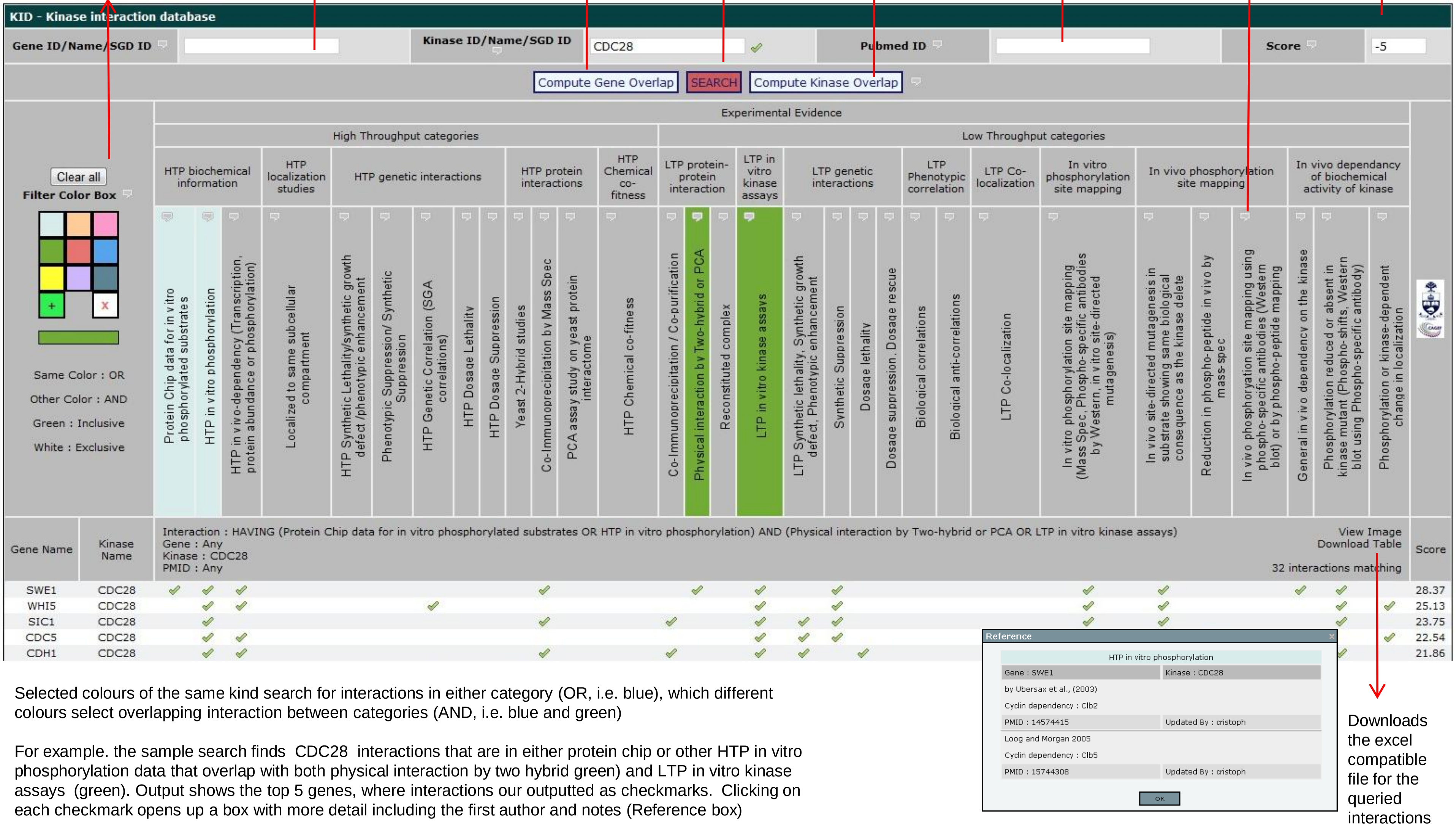

Downloads the excel compatible file for the queried interactions

Selected colours of the same kind search for interactions in either category (OR, i.e. blue), which different colours select overlapping interaction between categories (AND, i.e. blue and green)

For example. the sample search finds CDC28 interactions that are in either protein chip or other HTP in vitro phosphorylation data that overlap with both physical interaction by two hybrid green) and LTP in vitro kinase assays (green). Output shows the top 5 genes, where interactions our outputted as checkmarks. Clicking on each checkmark opens up a box with more detail including the first author and notes (Reference box)

Supplementary Figure 2:

HTP Categories

Biochemical Interactions
- *In vitro* Phosphorylation on a Protein Chip (Ptacek et al, 2006)
- HTP *in vitro* Phosphorylation (Ubersax et al, 2003; Dephoure et al, 2005; Holt et al, 2007)
- HTP *in vivo* Dependency [Phosphorylation Transcription, Protein abundance] (Usaite R. et al. 2009; Zaman S. et al, 2009, other studies)

Co-localization
- Localization of GFP-Tagged Proteins (excluding cytoplasm & nucleus) (Ho et al, 2006)

Genetic Interactions
- Synthetic Lethality (Costanzo et al, 2010; Collins et al, 2007; Fiedler et al, 2009; Jorgensen P et al, 2002; Tong et al, 2004; Pan et al, 2006; Wilmes et al, 2008, Sharifpoor et al, Submitted)
- Synthetic Suppression (Costanzo et al, 2010; Collins et al, 2007; Fiedler et al, 2009; Wilmes et al 2008; Sharifpoor et al, submitted)
- Dosage Lethality (Sharifpoor et al, Submitted)
- Dosage Suppression
- SGA Correlations (Costanzo et al, 2010, Sharifpoor et al, submitted)

Protein-Protein Interactions
- Y2H (Uetz et al, 2000, Ito et al, 2004) Core Dataset
- PCA Assay (Tarrasov et al, 2008)
- Co-IP by Mass Spectrometry (Ho et al, 2002; Gavin et al, 2006; Gavin et al, 2002; Krogan et al, 2006; Collins et al, 2007)

Chemical Co-fitness
- (Hillenmayer et al, 2008) Top 20 Chemically Co-fit Genes

LTP Categories

in vitro Kinase Assays

Co-localization

Genetic Interactions
- Synthetic Lethality
- Synthetic Suppression
- Dosage Lethality
- Dosage Suppression

Protein-Protein Interactions
- Co-IP or Co-purify
- Y2H or PCA
- Reconstituted Complex

Phenotypic Correlations
- Biological Correlation
- Biological Anti-correlation

in vitro Phosphorylation Site Mapping

in vivo Phosphorylation Site Mapping
- Site-Directed Mutagenesis
- Reduced Phospho-Peptides by Mass Spectrometry
- Phospho-Specific Antibodies, Western Blot

in vivo Dependency on Kinase Biochemical Activity
- General *in vivo* Dependency
- Phosphorylation Reduced or Absent in Kinase Mutant
- Phosphorylation Dependant Change in Localization

Supplementary Figure 3:

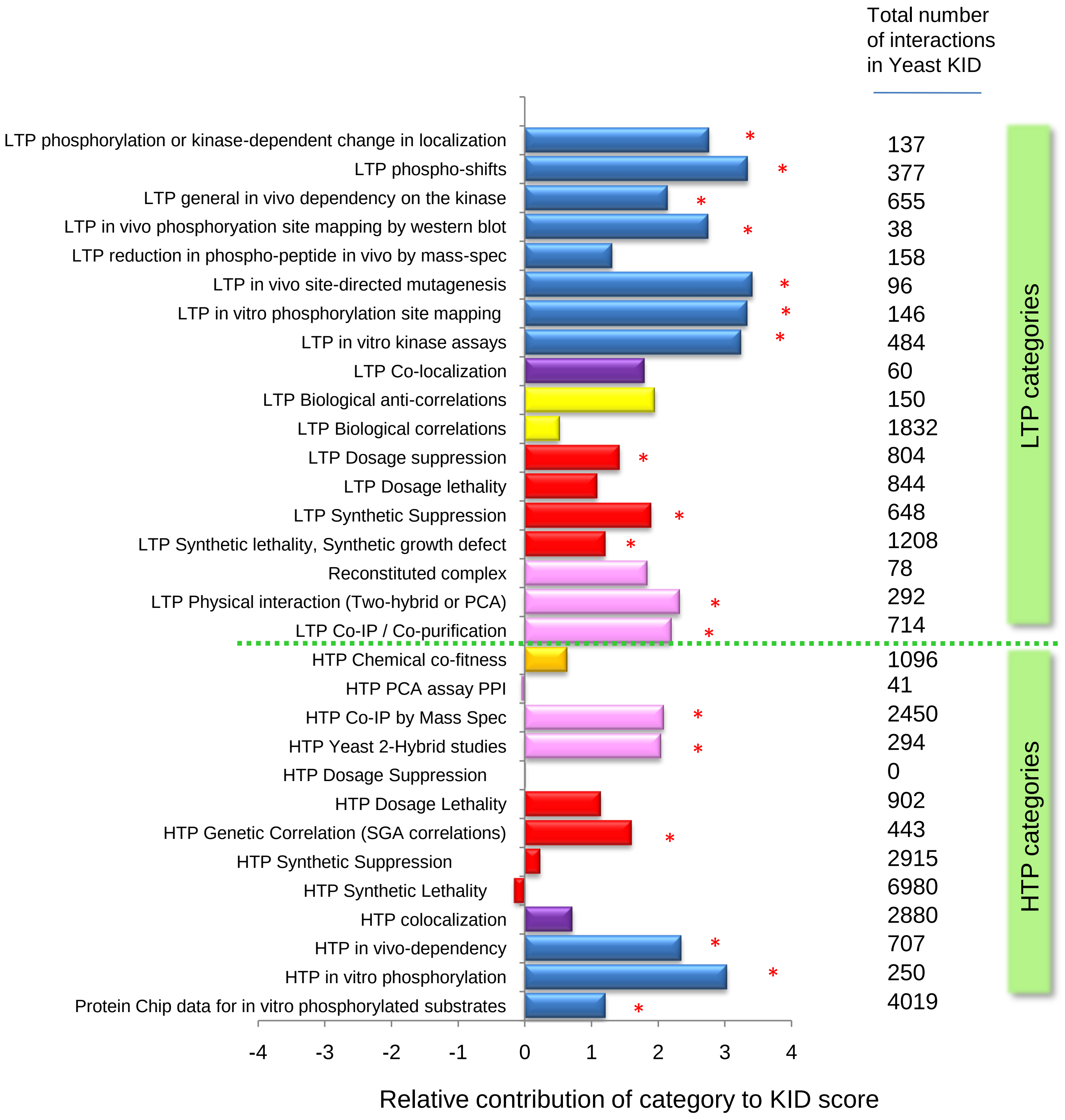

Supplementary Figure 4:

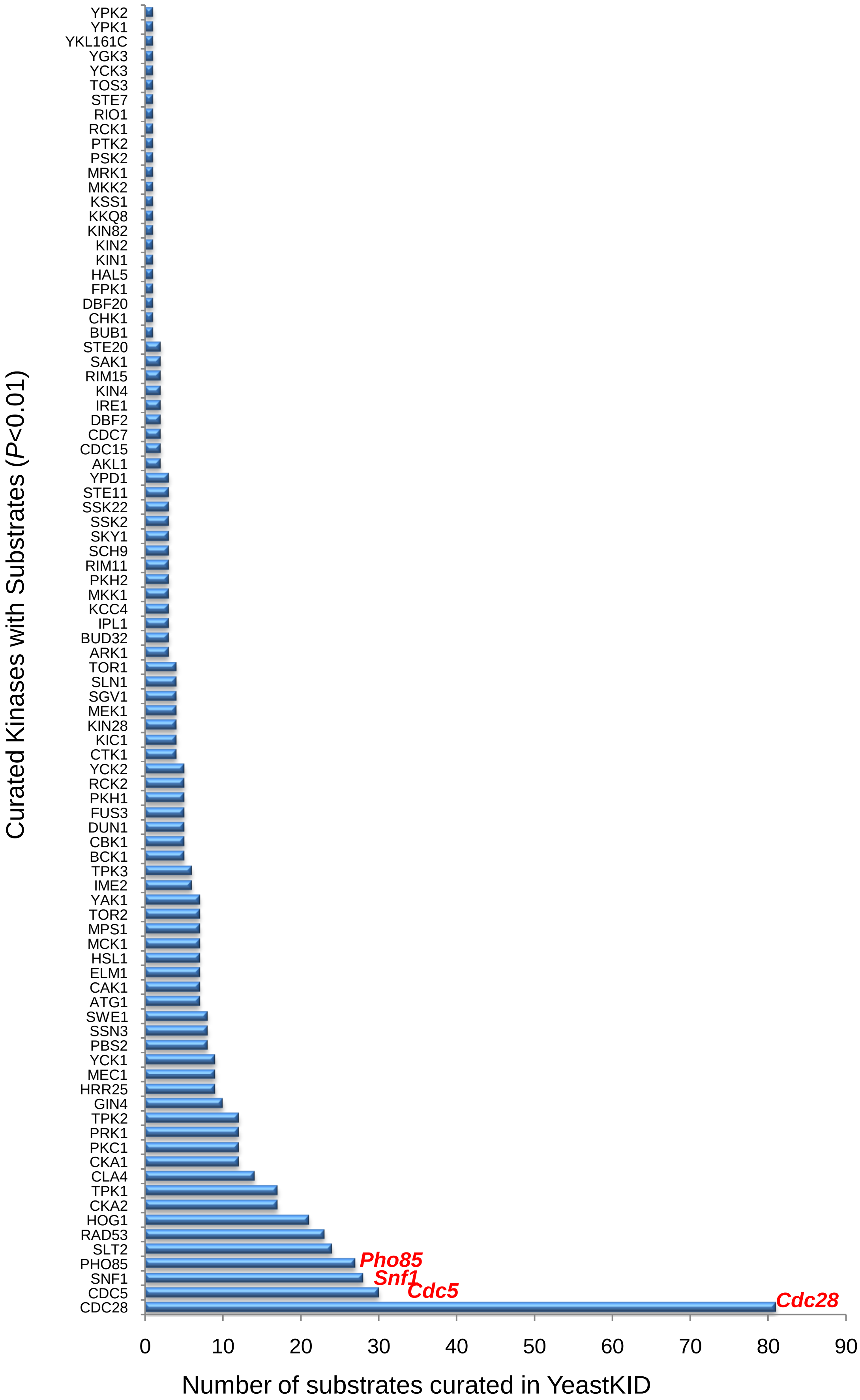

Supplementary Figure 5:

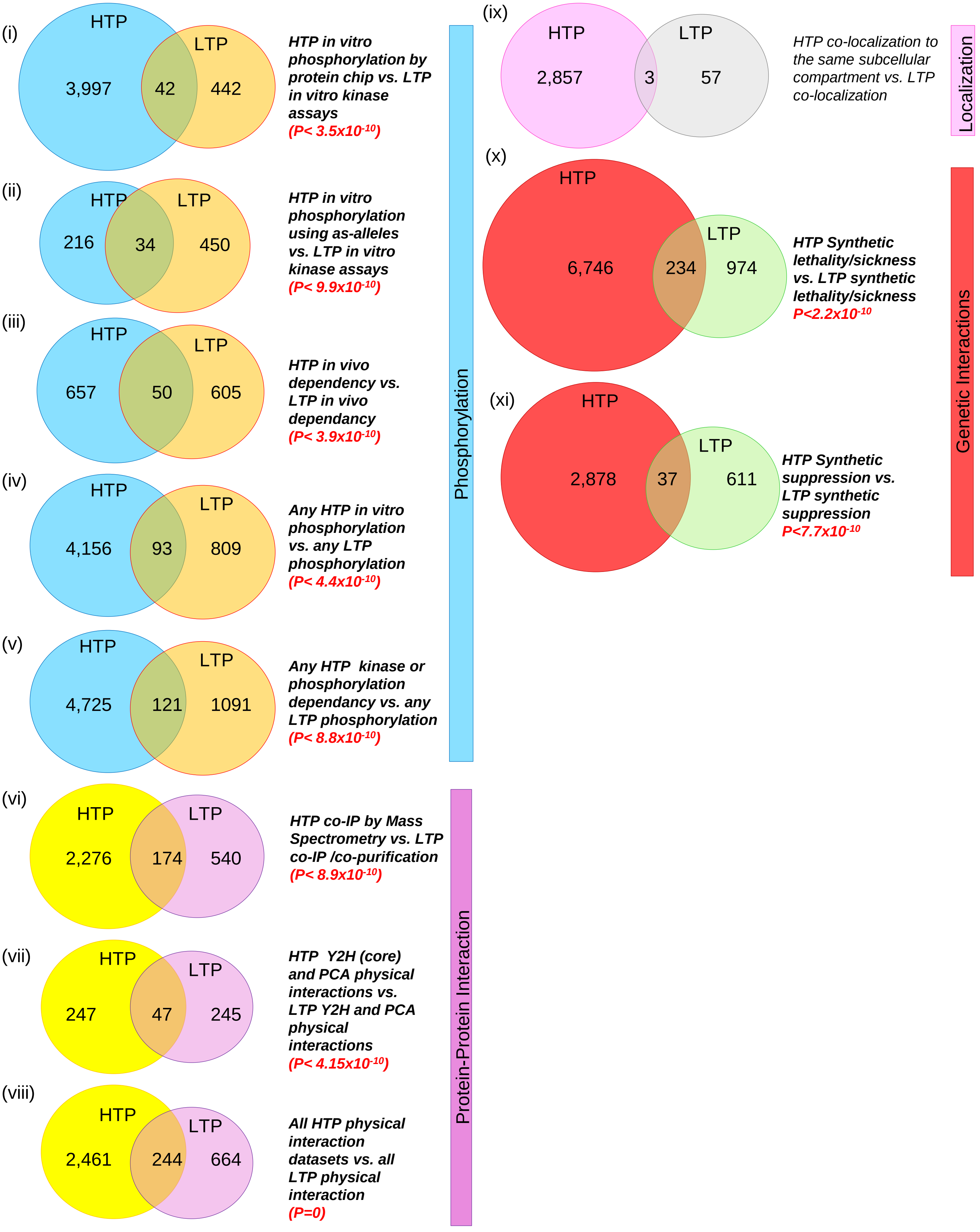

Supplementary Figure 6:

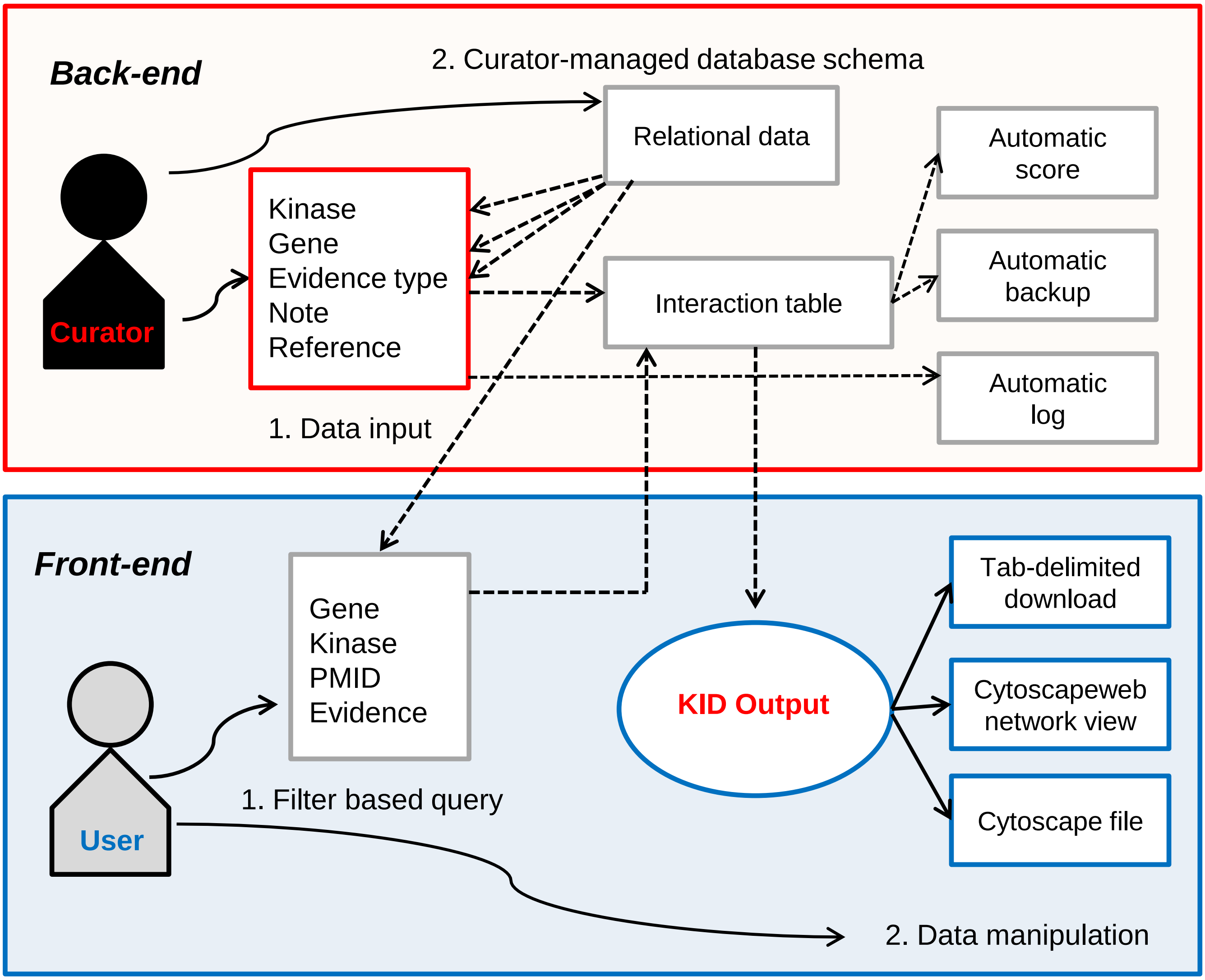

Supplement: Additional file 2 — Supplementary figures. Figure S1: Yeast KID user interface. A screen-shot of the Yeast KID homepage is shown. Experimental categories are hierarchically displayed and queried individually or in combination using the color box (left). Kinases, genes/proteins or PMIDs can be queried either individually or in combination, as single or multiple genes/proteins separated by commas or spaces. For multiple queries, overlapping interactions can be searched using the 'compute gene overlap' and 'compute kinase overlap' functions. Definition of each category and function is displayed by clicking on the small bubble icon for each category. See text for details. Figure S2: hierarchical division of Yeast KID categories. Chart showing 31 experimental categories hierarchically organized in three levels: 1) HTP and LTP categories (green); 2) overall subdivision of genetic, phenotypic, chemical, physical, cell biological or biochemical approaches (blue); 3) specific experimental assays (purple). Figure S3: KID weights of different LTP and HTP experimental categories. Relative contribution of different experimental categories in identifying the positive training kinase-substrate set. The bar graph indicates the contribution of each category to the KID score. Bars highlighted with a red star show significance when comparing categories relative to a random assignment of positive classes. The total number of interactions entered in each KID category is also presented. Red, genetic; pink, physical; blue, biochemical; yellow, phenotypic; purple, cell biological; orange, chemical. Figure S4: distribution of kinase substrates in Yeast KID. The graph shows the distribution of kinase targets reported in Yeast KID at the stringent cutoff (P < 0.01). Cdc28, Cdc5, Snf1 and Pho85 kinases have the largest number of targets in the literature. Thirty-seven curated kinases have no targets in Yeast KID at the stringent cutoff and are not represented on the graph. Figure S5: assessing the quality of HTP [file gb-2011-12-4-r39-S2.PDF]
